# A biomechanical digital twin of Legg–Calvé–Perthes disease deformity

**DOI:** 10.1007/s11548-025-03553-4

**Published:** 2025-12-01

**Authors:** Luke G. Johnson, David R. Wilson, Kishore Mulpuri

**Affiliations:** 1 School of Biomedical Engineering, University of British Columbia, Vancouver, BC Canada; 2 BC Children’s Hospital Research Institute, Vancouver, BC Canada; 3 Department of Orthopaedics, University of British Columbia, Vancouver, BC Canada; 4 Centre for Aging SMART, 7/F 2635 Laurel Street, Robert H N Ho Research Centre, Vancouver, BC V5Z 1M9 Canada

**Keywords:** Legg–Calvé–Perthes disease, Biomechanics, Articular cartilage, Osteoarthritis, Digital twin model

## Abstract

**Purpose:**

The dynamic stress environment in the hip joint is thought to contribute to pain and osteoarthritis (OA) in people with Legg–Calvé–Perthes disease (LCPD) deformity, but is poorly understood, limiting clinical management options. The objective of this study was to develop and evaluate a patient-specific biomechanical “digital twin” model of LCPD to predict chondrolabral shear stress in dynamic and static loading scenarios.

**Methods:**

We produced a digital twin model of both hips in a patient with unilateral LCPD deformity using anatomical magnetic resonance imaging (MRI) and the *ArtiSynth* modeling platform. We evaluated the model’s sensitivity to changes in material properties and joint angles during a typical gait cycle, and verified its stress and femoral translation predictions against upright open MRI of the hip in high-flexion postures.

**Results:**

The model’s prediction of the highest chondrolabral shear stress during a gait cycle was 22–93% greater in the LCPD hip than in the unaffected hip. The model was sensitive to changes in material parameters and joint angles, but could accurately reproduce femoral translation and expected stress distribution in extreme static postures.

**Conclusion:**

This study demonstrates the importance of both dynamic motion and morphology in the stress environment of highly aspherical hip joints. Although some challenges remain, digital twin models are a promising tool to study the long-term outcomes of LCPD, and could be applied in future to aid clinical management.

**Supplementary Information:**

The online version contains supplementary material available at 10.1007/s11548-025-03553-4.

## Introduction

Legg–Calvé–Perthes disease (LCPD) is a pediatric hip condition that often results in a permanent residual deformity of the hip joint after healing [1]. Characteristic features of LCPD deformity include femoral head asphericity and flattening, joint incongruency, and acetabular dysplasia [2]. Residual LCPD deformity contributes to increased pain, reduced quality of life, and a high incidence of osteoarthritis (OA) during adulthood [3, 4]. The most severe cases are indicated for surgical intervention to reduce pain, improve function, and delay the need for total hip arthroplasty [5, 6]. In a recent survey of adults who had LCPD as children, 6.4% of male and 12.3% of female respondents reported having surgery after the age of 18 years [3].

There is a need to better understand the patient-specific mechanical environment in hips with LCPD deformity to choose treatments and plan surgeries. A primary goal of surgical treatment is to reduce the potential for bony impingement between the femur and the acetabular rim [5]. Impingement is known to cause OA by inducing high mechanical stresses in the local cartilage, labrum, and chondrolabral boundary [6]. Because the location of impingement cannot easily be derived from static preoperative imaging, current surgical guidelines recommend intraoperative range-of-motion inspection to diagnose functional pathology and inform intervention decisions [5]. The ability to predict chondrolabral stress concentrations during motion could shift decisions to the preoperative stage, potentially reducing intra-operative duration and reducing the rate of surgical failure, reported to be 16% in a review of follow-up studies [7]. Earlier knowledge, soon after healing, of the patient-specific stress environment could also inform preventative strategies such as gait and activity adjustments [8, 9] to delay the onset of symptoms and the need for surgery, and to improve surgical outcomes; surgical failure in addressing residual LCPD deformity is associated with preoperative pain scores and radiographic OA [10, 11].

Patient-specific biomechanical models of the hip joint (“digital twin” models) could be used to predict the impact of both LCPD deformity and clinical interventions on chondrolabral stress, in order to study and manage residual LCPD, but challenges remain in representing the condition’s heterogenous pathomorphology. Intra- and extra-articular impingement locations in LCPD have been predicted with a range-of-motion model based on patient-specific bony anatomy [12]. However, a binary impingement outcome provides limited insight into impingement severity, and chondrolabral stress may also be induced in non-impingement joint postures due to reduced articular contact area in highly aspherical and incongruent hip joints [13]. Finite element (FE) analyses have been used to study pathological stress distributions in the hip joint cartilage and labrum [14–17] but have often been restricted to static simulations [14, 15, 17] or have assumed simplified anatomical geometry [16]. A patient-specific dynamic model is needed to represent the interactions between the loading condition, joint motion, aspherical morphology and non-uniform cartilage thickness in LCPD [18]. Some inputs, such as cartilage material properties, can vary with age, disease and degeneration [19, 20]. Others, like hip joint angles during gait, are prone to measurement error [21, 22]. It is important to characterize the sensitivity of any model to these inputs to determine what assumptions may be made in future applications.

**Fig. 1:**
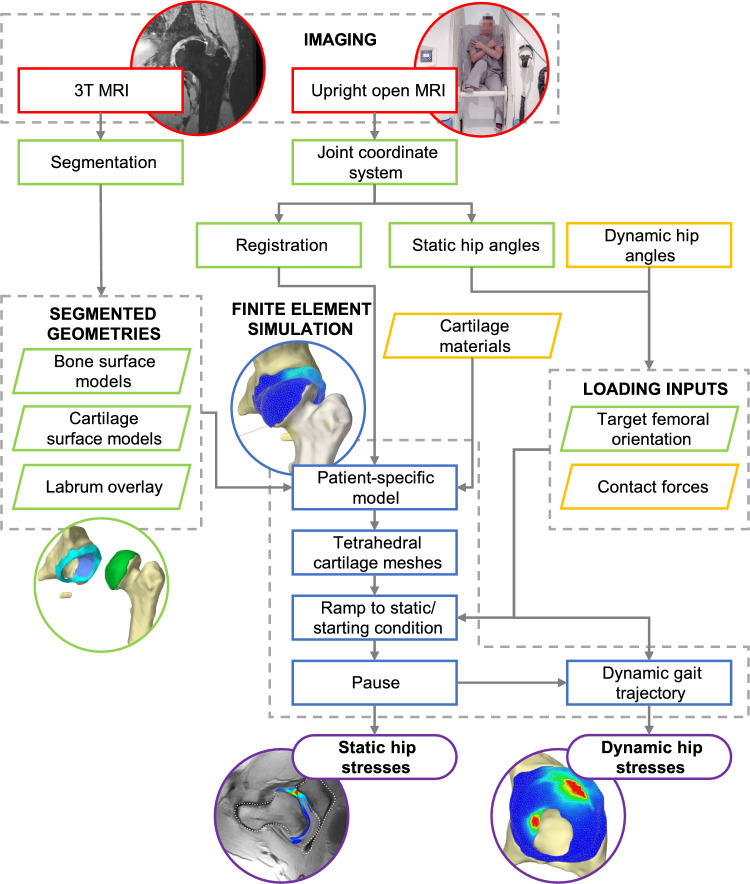
Flowchart of modeling steps. MRI data obtained from previous studies (red) were used to derive patient-specific input parameters (green) for the digital twin model. Where patient-specific data were not available, additional parameters were derived from the literature (yellow). The *ArtiSynth* digital twin model (blue) combined a rigid body representation of bone with FE simulation of the articular cartilage and labrum, resulting in maps of maximum-shear stress distribution during static and dynamic loading scenarios (purple)

Our objectives in this study were:

1. To develop a dynamic biomechanical digital twin model of a patient with severe unilateral LCPD deformity (Fig. 1), and
2. To evaluate the model’s sensitivity to physiologic variation in chondrolabral material properties and expected joint angle measurement errors during dynamic gait, and to evaluate the model’s ability to replicate real-world high flexion postures imaged using upright open magnetic resonance imaging (MRI).

## Materials and methods

### Patient and imaging

We retrospectively recruited one participant (F, 19 years old) with unilateral LCPD deformity (Stulberg grade IV [2]), who had taken part in previous MRI studies [23, 24].

An MRI scan of both hip joints was obtained using a GE Discovery MR750 3 T scanner (Waukesha, WI, USA), with a *MERGE* water excitation sequence (field of view = 320 mm, slice thickness = 1.4 mm, slice spacing = 0.7 mm, acquisition matrix = 512 × 384, echo time = 16.1 ms, repetition time = 30.4 ms, flip angle = 5°).

### Segmentation and mesh preparation

One rater (LJ) manually segmented the bone, articular cartilage, and labrum (including the transverse acetabular ligament) for each side using *3D Slicer* v5.3.0 [25]. Segmentation labelmaps were then exported as surface mesh stereolithography files with 20 iterations of Laplacian “joint smoothing” to remove marching cubes artifacts while retaining watertight boundaries between adjacent surfaces.

Final smoothing and decimation of the chondrolabral meshes was performed in *MeshLab* v2025.07 [26], using the following steps:

1. Quadric edge collapse decimation by 50%,
2. Taubin smoothing,
3. Isotropic explicit remeshing.

The collective aim of smoothing in *MeshLab* was to prepare the surface meshes for tetrahedralization by a) removing sharp edge features introduced by the joint smoothing algorithm, and b) encouraging faces with low aspect ratios (close to equilateral triangles). A full description of the parameters used for each step is available in Online Resource 1.

The target edge length for isotropic explicit remeshing was iteratively reduced by 10% until stress outputs changed by less than 5%, after which the mesh was considered converged. The final average edge length for the surface meshes was 0.72 mm.

### ArtiSynth model

We performed simulation using the open-source *Java*-based *ArtiSynth* platform (v3.8) [27] and the project code is available in Online Resource 1. Bony structures were defined to be rigid bodies, and the articular cartilage and labrum were modelled using tetrahedral meshes generated within *ArtiSynth* from the smoothed surface meshes.

#### Boundary conditions

**Fig. 2:**
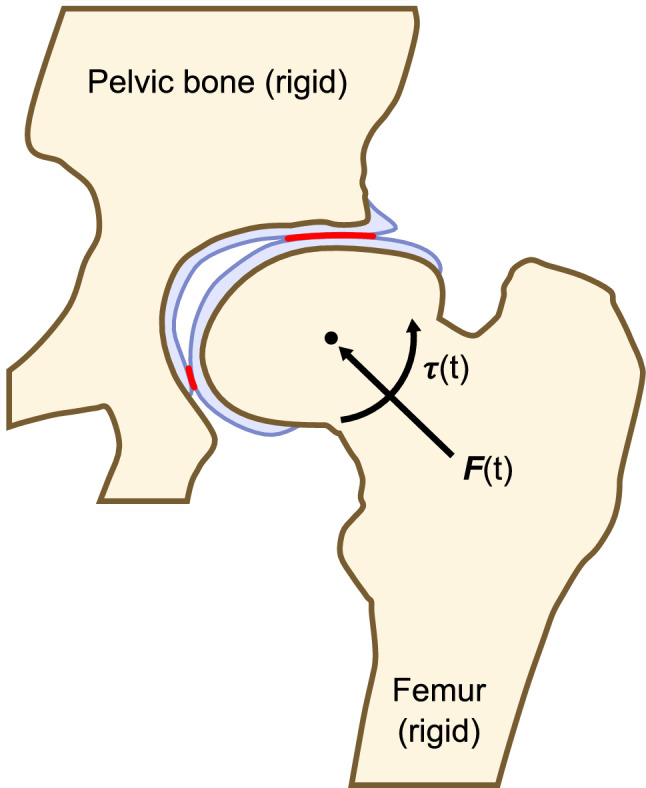
A simplified 2D diagram showing the boundary conditions and constraints in the digital twin model (affected side). The pelvic bone is fixed to the world coordinate frame. The femur is free to move with 6 degrees of freedom in response to force/torque inputs and constrained by frictionless contact (red) between the acetabular and femoral finite element meshes (blue) representing the joint’s articular cartilage and acetabular labrum

The pelvic bone was fixed, and the femur was free to move in all six degrees of freedom (Fig. 2). Nodes of FE meshes within 0.2 mm of the bone surface were considered part of the osteochondral boundary in order to accommodate vertex displacement during the mesh smoothing protocol. These nodes were attached to the bone, and frictionless contact was defined between the articulating femoral and acetabular finite element meshes (Fig. 2). Tests of typical friction coefficients for articular cartilage (µ = 0.001 and 0.01 [28, 29]) resulted in negligible changes to the model’s output at the expense of considerable increases in computation time (Online Resource 1).

Joint rotations were input as anatomical rotations (flexion, adduction, and internal rotation) using a standard hip joint coordinate system [28] and we produced a class of methods to convert between anatomical angles and 3D rotation matrices in *ArtiSynth.* User-defined joint rotation trajectories were applied to the femur via a simple proportional-derivative torque control loop and the corresponding joint contact forces were applied directly to the femoral bone at the femoral head center (Fig. 2).

#### Material properties

We modeled articular cartilage as a Neo-Hookean hyperelastic material, with material parameters for dynamic and static loading derived from Park et al. (2004) and Athanasiou et al. (1995) respectively (Table 1) [20, 29].

**Table 1:** Neo-Hookean hyperelastic material parameters for articular cartilage and labrum matrix models

*Anatomy/material scenario*	Lamé parameters		Equivalent linear elastic parameters at zero strain	
	λ (MPa)	μ (MPa)	E (MPa)	ν
*Articular cartilage*				
Dynamic (baseline)	533	5.4	16.1	0.495
Dynamic (stiffer)	1348	13.6	40.7	0.495
Dynamic (osteoarthritic)	113	1.14	3.4	0.495
Static	41.4	0.42	1.25	0.495
*Labrum matrix*				
Dynamic (all)	210	0.42	1.26	0.499
Static	26.1	0.052	0.157	0.499

We represented the labrum as a transversely isotropic hyperelastic material region of the acetabular FE mesh, consisting of a Neo-Hookean matrix material base and a directional overlay representing the circumferential fiber orientation. We derived equilibrium matrix and fiber material parameters from Ferguson et al. (2001) [30]. In the absence of literature on the dynamic material properties of the acetabular labrum we assumed the matrix behaves similarly to articular cartilage, in that it has an instantaneous modulus 8 × greater than aggregate (equilibrium) modulus measured from the same sample (Table 1) [31]. We used custom *Java* code to calculate the fiber direction at each FE node from the labral geometry during the *ArtiSynth* model’s instantiation (Online Resource 1). The fiber overlay material was based on the passive properties of an *ArtiSynth* GenericMuscle material [32], with a toe region response described by an exponential stress coefficient of 0.051 MPa and uncrimping factor of 36, an exponential/linear transition stretch of 1.103, and a straightened fiber modulus of 74.7 MPa [30].

#### Simulation and outputs

Simulation was performed using *ArtiSynth*’s semi-implicit Newmark integrator with a simulation time step size of 0.002 s. FE meshes were represented with lumped-mass linear tetrahedral elements [27]. The output of the simulation was peak maximum-shear stress. At each sample time step (t_s_ = 0.01 s), the stress for each FE node was calculated, and the highest values in the femoral cartilage and acetabular cartilage/labrum were recorded.

### Dynamic gait simulation

We evaluated the dynamic performance of the digital twin model by simulating a typical level gait cycle in both hips, with the unaffected contralateral hip representing normal anatomy. We obtained joint contact force and angle trajectories for a typical level gait cycle from the literature [33, 34] and contact forces were scaled to the participant’s body weight (BW) for simulation.

#### Sensitivity analysis

We assessed the dynamic model’s sensitivity to expected variation in material properties and variation in joint angles due to motion capture error. We tested two material scenarios: one with a stiffer estimate of material parameters as used in some previous studies [14, 15] and one with more compliant properties representative of osteoarthritic cartilage [19] (Table 1). Additionally, we simulated four motion capture error scenarios, adding a constant ± 5° of flexion or ± 10° of internal rotation to the joint angle trajectory, representative of expected errors in marker-based motion capture systems during low-flexion activities such as gait [21, 22].

To test the repeatability of the baseline model, we ran it for two consecutive gait cycles and compared the peak stress output across the first and second cycles. To quantify differences between repeated gait cycles, between sensitivity analysis scenarios and between the affected and contralateral hips, we calculated the mean absolute percentage error (MAPE) for the whole gait cycle, separately for the femoral and acetabular meshes on the affected and contralateral sides:

$$\mathrm{MAPE} = \frac{100}{n}\sum_{i=1}^{n}\frac{\left|{x}_{i,\mathrm{test}}-{x}_{i,\mathrm{baseline}}\right|}{{x}_{i,\mathrm{baseline}}}$$

where $n$ is the number of samples recorded. We also calculated the mean percentage error (MPE) to determine whether different scenarios resulted in a consistent over- or underestimate of peak stress compared to the baseline model:

$$\mathrm{MPE} = \frac{100}{n}\sum_{i=1}^{n}\frac{{x}_{i,\mathrm{test}}-{x}_{i,\mathrm{baseline}}}{{x}_{i,\mathrm{baseline}}}$$

The value of MPE always lies in the range ± MAPE. Positive and negative values MPE indicate test scenarios resulting in higher and lower peak stresses on average than the baseline simulation. If MPE =  + MAPE, the test scenario peak stress is higher than baseline at every sample time point, and lower at every time point if MPE = − MAPE.

### Static high flexion simulation

We evaluated our digital twin model’s ability to accurately predict expected regions of high stress and femoral translations during impingement by reproducing static neutral and high flexion postures that had been imaged in an upright open MRI scanner (MROpen, ASG Superconductors, Genoa, Italy) [23].

The upright open MRI protocol included axial scans of the iliac crests and the distal femur in the supine posture, and sagittal and oblique scans of the hip joint in all postures. The axial and supine sagittal images provided the anatomical landmarks used to define each hip’s joint coordinate system [28]. We obtained transformation matrices for the pelvis and femur between each posture by rigid registration of the bones segmented from each sagittal image and converted these transformations to anatomical rotations for the simulation.

To simulate each posture (supine; seated; supine with high flexion, adduction, and internal rotation (FADIR); and seated FADIR), we first applied a constant force of 30% BW, typical of passive supine or seated postures [34], normal to the acetabular opening. The position and orientation of the oblique imaging planes, used to visualize anterior impingement in each posture in the upright open MRI, were obtained from image metadata. Custom *MATLAB* (version R2024a) code, available in Online Resource 1, was used to transform these planes to the starting femoral orientation, after which they were fixed to the femoral reference frame. The femur was then slowly rotated to the posture of interest and allowed to equilibrate, before taking a cross-section of the model in the oblique imaging plane for that posture and superimposing the cross-section stress map and bone outlines onto the corresponding MRI slice. From these composite images, we qualitatively assessed how well the model’s femoral and pelvic bone outlines matched the real-world MRI, and whether the model predicted stress concentrations corresponding with visible impingement locations.

## Results

### Dynamic gait simulation

**Fig. 3:**
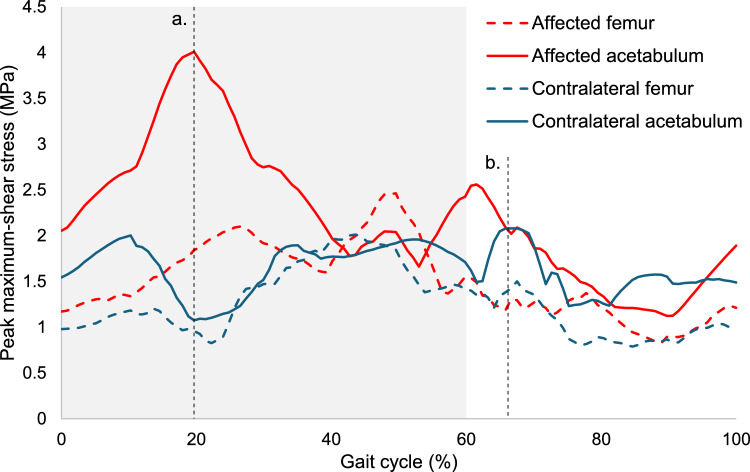
Trajectory of peak maximum-shear stress measured in the femoral cartilage (dashed lines) and acetabular cartilage and labrum (solid lines). The hip with LCPD deformity is shown in red, with the unaffected contralateral hip in blue. The shaded portion of the chart represents the stance phase of gait. The overall peak shear stress in the affected (**a**) and contralateral (**b**) acetabula are indicated with vertical dotted lines and correspond to the stress maps in Fig. 4. LCPD, Legg–Calvé–Perthes disease

Peak maximum-shear stress was substantially higher in the LCPD-affected acetabulum compared to the contralateral acetabulum, particularly during the early and middle stance phases of gait (Fig. 3). The highest stress measured in the affected femur was 2.46 MPa, compared to 2.01 MPa at in the unaffected femur—an increase of 23%. In the affected acetabular cartilage and labrum, the highest stress was 4.01 MPa, 93% higher than 2.08 MPa on the unaffected side. The MAPE of the affected compared to the contralateral side across the whole gait cycle was 26.9% in the femoral cartilage (MPE 24.1%) and 47.9% in the acetabulum (42.2%).

**Fig. 4:**
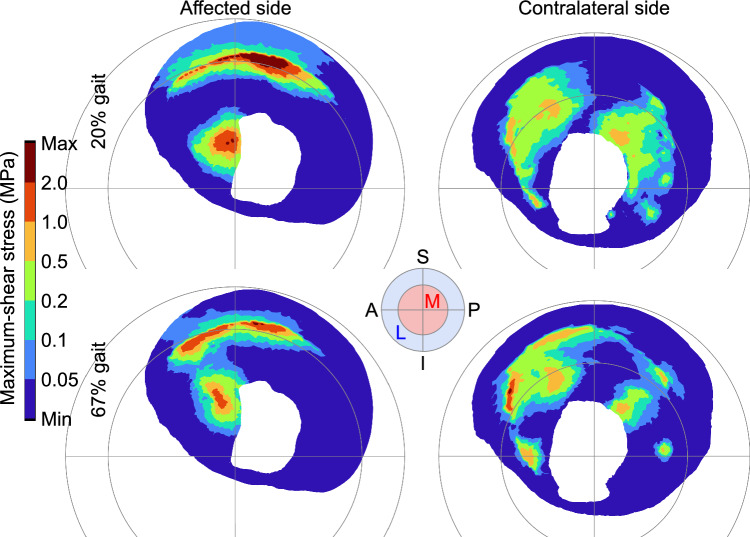
Colormaps showing the distribution of maximum-shear stress on the articulating surfaces of the affected (left) and contralateral (right) acetabula at 20% (top) and 67% (bottom) gait, the respective time of the model’s prediction of peak stress for each hip. Hips are projected with the anterior side to the left and medial at the center. The larger medial region in in the affected hip is caused by the lateral displacement of the joint coordinate system origin due to a shallow, dysplastic acetabulum

The overall peak shear stress was measured in the affected and contralateral acetabula at 19.7% and 66.7% gait respectively (Fig. 3) and 49.6% and 43.6% gait in the affected and contralateral femoral cartilage. At peak, shear stress was concentrated in the superior and medial-anterior regions of the affected acetabulum and in the anterior-superior region of the contralateral acetabulum (Fig. 4).

In our repeatability assessment, differences in peak stress between the two consecutive cycles were low, with MAPE ranging from 0.50% to 0.73%, and a MPE ranging from −0.50% to 0.14%. Model predictions of peak stress were more sensitive to errors in material stiffness than to motion capture error (Table 2).

**Table 2:** Sensitivity of the digital twin model to changes in material parameters and motion capture error, represented by the mean absolute relative error (MAPE) and mean relative error (MPE) of peak maximum-shear stress across all sample time points (n = 118) in a simulated gait cycle. If MAPE and MPE have the same magnitude, all errors are in the same direction

Sensitivity analysis scenario	MAPE (MPE) relative to baseline model (%)			
	Affected hip		Contralateral hip	
	Femoral	Acetabular	Femoral	Acetabular
Increased material stiffness	76.9 (76.9)	85.4 (85.4)	106.4 (106.4)	115.6 (115.6)
Reduced material stiffness	60.6 (− 60.6)	62.7 (− 62.7)	63.5 (− 63.5)	69.9 (− 69.9)
5° added flexion	12.0 (− 3.5)	6.6 (0.2)	13.6 (4.7)	18.1 (6.7)
5° added extension	11.0 (7.9)	7.5 (2.6)	9.3 (3.1)	22.8 (− 0.9)
10° added internal rotation	14.7 (− 2.2)	16.7 (− 10.0)	13.1 (9.9)	13.1 (3.4)
10° added external rotation	15.1 (2.0)	13.6 (13.3)	24.1 (13.5)	29.7 (21.8)

### Static high flexion simulation

**Fig. 5:**
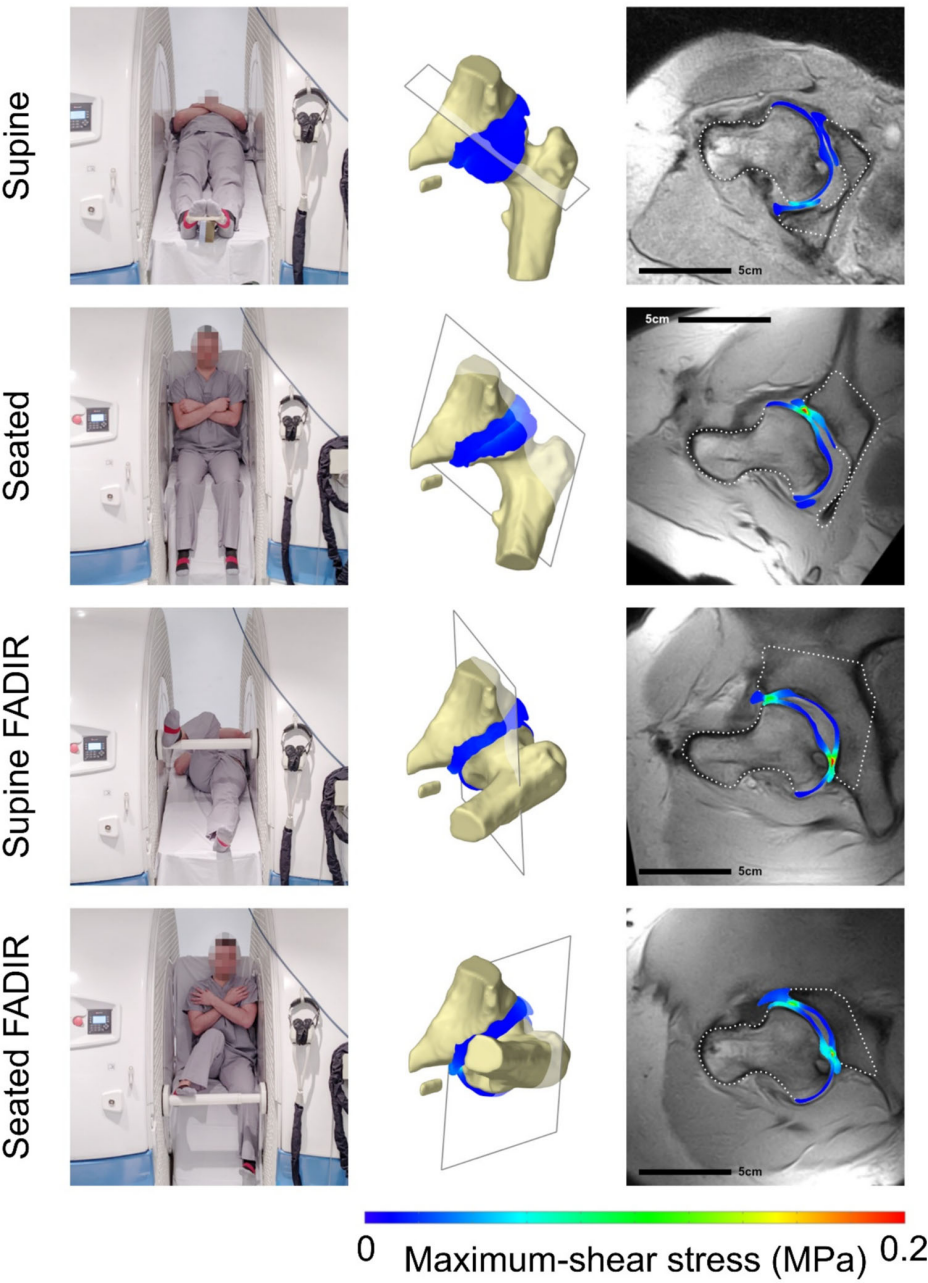
Results from static simulation of real-world high-flexion postures. Left column: the participant’s affected hip was scanned in four postures in an upright open MRI scanner (demonstrated by a volunteer). Middle column: these postures were reproduced in the digital twin model and a cross-section was taken in the imaging plane. Right column: chondrolabral stress maps from the digital twin were superimposed on the corresponding MRI slice. MRI, magnetic resonance imaging; FADIR, flexion, adduction and internal rotation

In our simulation of static, high flexion postures, the digital twin model predicted high stress regions in locations where anterior impingement was apparent on MRI: the neutral seated, supine FADIR and seated FADIR postures (Fig. 5). Additional high stress regions were visible in the posterior acetabular rim in the latter two postures.

## Discussion

In this study, we created and evaluated a digital twin biomechanical model of the hip joint and demonstrated its utility in estimating static and dynamic chondrolabral maximum-shear stress in a patient with unilateral LCPD deformity.

The results from our digital twin model provide insight into why LCPD hips with residual deformities may be prone to OA, even if gait mechanics are normal. During simulation of a typical gait cycle, the highest stress predicted in the affected side was almost 2 × greater than the highest predicted in the contralateral side. The peak stress in the unaffected hip varied less across the gait cycle compared to the LCPD hip. The applied joint contact force varied by a factor of 5.63 (41.2% to 232.1% BW), whereas the peak stress in the unaffected and LCPD acetabula varied by factors of 1.94 and 3.57 respectively. Load is distributed through materials by a combination of shear and hydrostatic stress components, carried primarily through the solid matrix and fluid portion of cartilage respectively [35]. The results from this patient suggest that LCPD deformity causes a larger proportion of load to be carried by shear stress, resulting in microstructural damage to the matrix and macrostructural cartilage delamination and labral tears [35].

**Fig. 6:**
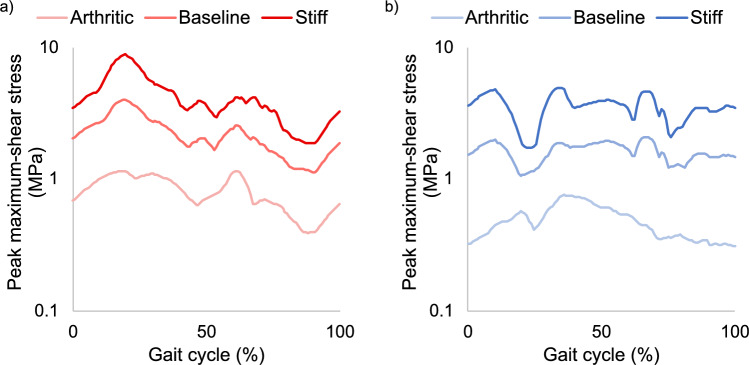
Changes in peak maximum-shear stress (log scale) across the gait cycle in response to increasing chondrolabral material stiffness parameters in **a** the LCPD-affected acetabulum (red) and **b** the unaffected acetabulum (blue)

Our digital twin model is highly sensitive to changes in material properties, so we need accurate measures of chondrolabral properties and their response to age, disease and degeneration. We derived our baseline cartilage material parameters from the “minimum” (zero-strain) modulus (16.1 MPa) measured during 1 Hz cyclic compressive loading by Park et al. [29]. An alternative approach in past work has been to take the average of the minimum and maximum moduli (40.7 MPa) [14, 15], which we replicate in our “stiffer cartilage” sensitivity analysis scenario. We believe our baseline estimate is more representative of healthy cartilage, as the zero-strain modulus is more consistent with the instantaneous modulus measured from human cartilage in other work [19, 36]. Peak stress in our model appears to vary proportionally with cartilage stiffness in both LCPD and normal hips (Fig. 6). In patients with unilateral residual LCPD it is reasonable to assume that cartilage properties are similar in each hip prior to long-term degradation [24], so we expect the model's predictions of the location and relative magnitude of stress concentrations to be useful even if there is substantial error in the chosen material stiffness.

The digital twin model is also sensitive to errors in joint rotations, so care should be taken to ensure the accuracy of this input. Unexpectely, the contralateral side was more sensitive to systematic changes in flexion and internal rotation than the affected side, with a MAPE of up to 30% in response to expected errors from marker-based motion capture systems. In particular, an added 10° of external rotation tended to increase the estimated peak shear stress across the gait cycle, with a positive MPE of 21.8%, about half the size of the effect of LCPD deformity in the baseline model (MPE = 42.2%). This sensitivity illustrates a potential limiting factor for the utility of biomechanical digital twin models in a clinical context. Conversely, these results also suggest gait or activity adjustment could be an effective tool to manage joint stress and slow cartilage degradation in LCPD [8, 9]. For example, adding 10° of internal rotation resulted in an MPE of −10.0% in the affected acetabular cartilage, indicating that a toe-in gait could be used to reduce peak shear stress throughout the gait cycle.

Our static simulation of high flexion postures and comparison with real-world MRI data provides verification of the digital twin model’s physiologic accuracy, a strength of this study. The relationship between anterior hip impingement visible on upright open MRI and increased acetabular contact pressure has been validated using instrumented cadaver hips [37]. In previous work, we assessed that this participant’s LCPD-affected hip was impinging in every posture except the neutral supine posture [23]. The digital twin model predicts stress concentrations at the anterior acetabular rim in these postures (Fig. 5) consistent with the expected stress distribution. In our model, femoral translation was only constrained by articular contact. Our static simulation demonstrates that this approach is appropriate, as the model’s femoral position closely matches the real-world MRI even in high flexion postures where the femur and acetabulum are highly incongruent (Fig. 5).

The results from our model are consistent with existing patient-specific models of normal and LCPD hips. In a quasi-static simulation of the peak hip loading condition during late stance, Ng et al. [15] reported a maximum acetabular shear stress of 4.0 MPa in a healthy adult male control. The “stiffer cartilage” scenario of our dynamic gait model was chosen to represent the previous work’s material properties and predicted a peak shear stress of 3.93 MPa at the corresponding point of the gait cycle (50% gait). However, the overall peak occurred at 34% gait and was 25% higher (4.96 MPa), demonstrating the contribution of both loading and motion to shear stress during activity. A limitation of this comparison is that it is between patient-specific analyses of two different individuals, so the influence of factors such as inter-patient variation, particularly sex-based anatomical differences, and methodological differences, such as the material representation of bone, is unclear. Our finding of increased acetabular stress in the LCPD hip compared to the contralateral control hip was consistent with a FE model study’s finding of increased stress in a LCPD hip’s acetabular cartilage compared to the contralateral hip’s acetabular cartilage during static two-leg stance (von Mises stress of 5.0 MPa and 3.6 MPa respectively [17]). This study was not directly comparable to our own due to the heterogeneity of LCPD and differences in stress reporting.

The primary strength of the present model is the combination of patient-specific anatomy and dynamic simulation. The results from our level gait demonstration showed that both joint contact load and joint orientation have a large influence on chondrolabral stress: the highest stresses were observed at different points in the gait cycle for the affected femoral (49.6% gait), contralateral femoral (43.6%), affected acetabular (19.7%) and contralateral acetabular (66.7%) cartilage and labrum (Fig. 3). Past simulations of level gait have approximated this activity with static loading representing single positions at heel strike, mid-stance, and during terminal stance [14, 15]. Using the same methodology in this patient would have provided a misleading impression of the peak stress experienced in the affected hip, emphasizing the need to consider both 3D morphology and motion in highly aspherical LCPD hips.

We believe that dynamic digital twin models could play an important role in clinical management of residual LCPD and similar hip joint pathomorphology. Stress maps derived from patient-specific activities and compared with proposed activity adjustments could help physiotherapists determine which adjustments would be the most effective at reducing shear stress concentrations. Recent work has shown that using gait adjustment to reduce medial compartment load in the knee is effective at slowing the rate of cartilage degradation and OA onset [8, 9]. If surgical remodeling of the affected hip joint is indicated to improve function and reduce severe pain, a digital twin model could be used to preview the effectiveness of proposed surgical plans and reduce the time required for intraoperative inspection [5].

One key limitation of this study is that we lack patient-specific gait information for our participant. Although literature estimates of typical gait angle and loading trajectories are sufficient to demonstrate the feasibility of an LCPD digital twin model, our results may not be representative of the actual shear stress environment in this participant’s hip during walking. Patients with LCPD are known to modify their gait in response to deformity or pain [38, 39], so future implementations should incorporate patient-specific gait parameters. However, the presence of elevated shear stress in the present model’s LCPD hip during a “normal” gait trajectory, along with the sensitivity of stress predictions to changes in joint orientation, illustrates the motivation for LCPD patients to alter their gait patterns. Direct validation of the model’s stress predictions is not feasible. Validation of hip FE models is typically performed using instrumented cadaveric samples [40, 41], which would be impractical to obtain in LCPD deformity.

We have developed a biomechanical digital twin model of LCPD deformity that combines, for the first time, patient-specific hip anatomy with fully dynamic simulation of activities. The model captures the complex interaction between morphology, loading and motion in aspherical hips. While the model is sensitive to errors in material properties and joint angles, the differences would be small within patients compared to the large effect of LCPD deformity on peak maximum-shear stress. This model has applications in studying and managing patient-specific factors that lead to early-onset OA.

## Supplementary Information

Below is the link to the electronic supplementary material.

Supplementary file1 (PDF 442 KB)
