# Supplementary material for: A biomechanical digital twin of Legg–Calvé–Perthes disease deformity
Source: Int J Comput Assist Radiol Surg. 2025 Dec 1;21(4):735–45. doi: 10.1007/s11548-025-03553-4 (PMC13194310; doi:10.1007/s11548-025-03553-4)
Supplement: Supplementary file 1 — Supplementary file1 (PDF 442 KB) [file 11548_2025_3553_MOESM1_ESM.pdf]

Online supplemental material

This document contains supplemental material for the article “A biomechanical digital twin of Legg–Calvé–Perthes disease deformity” (Luke G. Johnson[1,2], David R. Wilson[3]†, Kishore Mulpuri[2,3]; International Journal of Computer-Assisted Radiology and Surgery)

[1]School of Biomedical Engineering, University of British Columbia, Vancouver, British Columbia, Canada
[2]BC Children’s Hospital Research Institute, Vancouver, British Columbia, Canada
[3]Department of Orthopaedics, University of British Columbia, Vancouver, British Columbia, Canada

† Correspondence: David R. Wilson, Centre for Aging SMART, 7/F 2635 Laurel Street, Robert H N Ho Research Centre, Vancouver BC, V5Z 1M9; Email: david.wilson@ubc.ca; Phone: +1 (604) 675 2584

Surface mesh preparation steps

In *3D Slicer,* anatomical segmentations were converted from labelmaps to surface meshes by the marching cubes algorithm followed by 20 iterations of Laplacian “joint smoothing”. The resultant meshes are free of marching cubes step artifacts but often have sharp edge features where smoothing of the cartilage meshes was constrained by the need to retain a watertight boundary with the corresponding bone mesh.

Surface meshes were imported into *MeshLab* for additional preparation prior to tetrahedralization in *ArtiSynth* (Fig. S1). The goals were to remove any sharp edge features, to reduce the number of surface triangles with high aspect ratios, and to control the final finite element mesh density via the average edge length of the smoothed surface mesh.

Quadric edge collapse decimation

This standard mesh decimation algorithm was used to reduce the face count by 50%. The intended effect was to increase the relative prominence of edge features, improving the performance of the subsequent Taubin smoothing step.

Parameters used (bolded values are changed from default):

- Target number of faces: (leave – overridden by percentage reduction)
- Percentage reduction (0..1): **0.5**
- Quality threshold: **0.8**
- Preserve Boundary of the mesh: unchecked
    - Boundary preserving weight: (leave – N/A)
- Preserve normal: unchecked
- Preserve topology: unchecked
- Optimal position of simplified vertices: checked
- Planar simplification: unchecked
    - Planar simp. weight: (leave – N/A)
- Weighted simplification: unchecked
- Post-simplification cleaning: checked
- Simplify only selected faces: unchecked

Taubin smoothing

This smoothing step is used to smooth out the sharp edge features, reducing the likelihood of poor-quality tetrahedra being generated in *ArtiSynth*.

Parameters used (all default):

- Lambda: 0.5

- Mu: -0.53
- Smoothing steps: 10
- Affect only selected faces: unchecked

Isotropic explicit remeshing

This stage has two goals: to control the average size of surface mesh faces and therefore the final finite element mesh density, and to do so in a way that ensures good quality, low aspect ratio faces.

Parameters used (bolded values are changed from default):

- Iterations: 10
- Adaptive remeshing: **checked**
- Remesh only selected faces: unchecked
- Target length:
    - Abs: **[your desired target length]**
    - %: (leave – overridden by abs)
- Crease angle: **45**
- Check surface distance: **checked**
- Max surface distance:
    - Abs: **0.1**
    - %: (leave – overridden by abs)
- Additional steps (refine, collapse, edge-swap, smooth, reproject): all checked

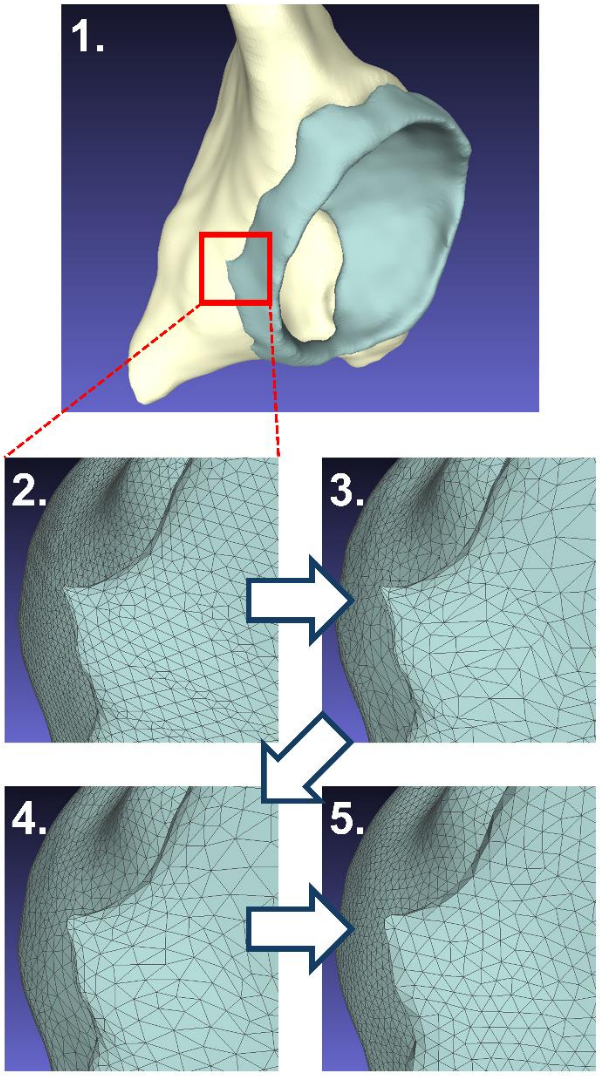

Fig. S1 Mesh smoothing steps in *MeshLab*. 1) Surface meshes of the affected pelvis and acetabulum from *3D Slicer*. 2) A closer view of a sharp edge feature on the original acetabular mesh and after 3) quadric edge collapse decimation, 4) Taubin smoothing, and 5) isotropic explicit remeshing.

Mesh density and convergence

We chose the output variable of interest for convergence to be mean percentage error (MPE) in peak shear stress across a typical gait cycle, compared to the previous stage of mesh refinement. The target surface mesh edge length was chosen when a subsequent 10% reduction in edge length resulted in an MPE of less than 5% (bolded values in the table below) in the femoral and acetabular finite element meshes in both the affected and contralateral hips.

Target surface mesh edge length (mm)	MAPE (MPE) relative to previous mesh refinement stage (%)			
	Affected hip		Contralateral hip	
	Femoral	Acetabular	Femoral	Acetabular
1.50	N/A	N/A	N/A	N/A
1.35	6.07 (**0.39**)	7.37 (-**1.92**)	8.83 (7.52)	11.04 (**1.87**)
1.22	7.45 (5.12)	6.23 (**3.37**)	12.80 (-10.55)	9.12 (**3.96**)
1.09	12.09 (-10.52)	8.78 (**3.79**)	7.64 (5.43)	26.95 (26.95)
0.98	9.08 (6.10)	15.05 (12.42)	6.94 (-**1.55**)	5.52 (**2.25**)
0.89	11.94 (**0.00**)	7.48 (**1.77**)	11.70 (8.85)	7.97 (**0.83**)
0.80	11.43 (7.45)	7.95 (7.65)	5.28 (-**2.74**)	9.90 (6.40)
0.72	6.88 (**1.52**)	6.21 (5.79)	11.31 (7.69)	6.97 (-**4.12**)
0.65	5.35 (**1.70**)	4.86 (**4.60**)	7.73 (**4.74**)	10.74 (-**0.57**)

After tetrahedral meshing in *ArtiSynth*, the final average edge lengths in the finite element mesh ranged from 0.78-0.98mm.

ArtiSynth source code

ArtiSynth code for the model presented in this article is available via the following link:

https://codeberg.org/lukegjohnson/perthesdigitaltwin/src/commit/4f43bc7478ebcbd08877302b25df7f677514080d (commit dated 31st October, 2025).

Any subsequent updates are available on the repository's home page https://codeberg.org/lukegjohnson/perthesdigitaltwin

Sensitivity analysis: friction coefficient

Our digital twin model represented the articulation of the femoral and acetabular cartilage using frictionless contact. Comparison of the baseline model with scenarios including representative values of the friction coefficient for articular cartilage in joints results in very little change to the trajectory of peak maximum-shear stress across a gait cycle, at the expense of substantially increased simulation time.

Friction coefficient	MAPE (MPE) relative to baseline (frictionless) (%)				Increase in simulation duration relative to baseline (%)	
	Affected hip		Contralateral hip		Affected hip	Contralateral hip
	Femoral	Acetabular	Femoral	Acetabular		
0.001	0.17 (0.02)	0.23 (0.02)	0.14 (0.05)	0.19 (0.05)	93.2	198.4
0.01	0.96 (0.34)	1.29 (0.01)	0.81 (0.29)	1.03 (0.45)	90.4	177.0

Calculation of labral fiber directions

We calculated the direction of circumferential fibers in the labrum and transverse acetabular ligament using the following steps (illustrated in Fig. S2):

1. Fit a least-squares plane to all finite element nodes forming the labrum material overlay.
2. Define a sweep axis by fitting a circle to the node coordinates projected onto the plane. The sweep axis is perpendicular to the plane and passes through the circle's center coordinate.
3. For each node in the labrum overlay region, find the shortest line connecting the node and the sweep axis. The circumferential direction is mutually perpendicular to the sweep axis and this line.

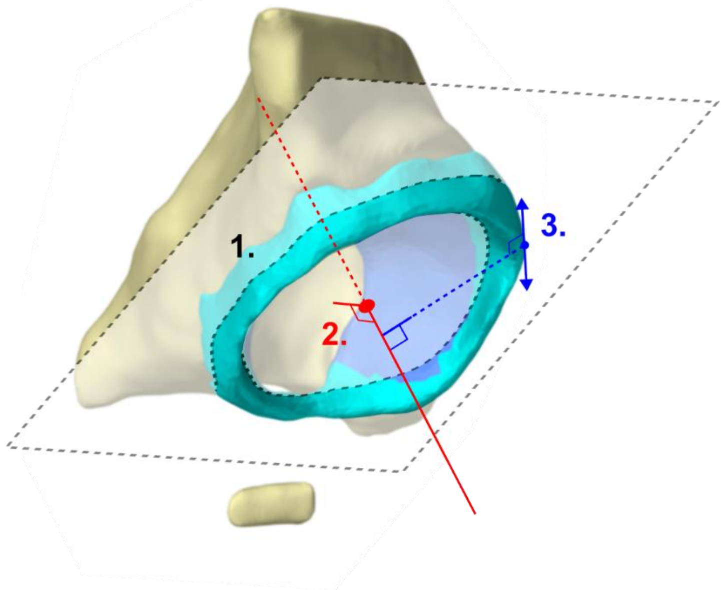

Fig. S2 Steps in the calculation of circumferential fiber directions.

The relevant section of code can be found in the source code file *PerthesDigitalTwinBase.java* between lines 246 and 273.
